# Supplementary material for: Prognostic Significance of EGFR, HER2, and c-Met Overexpression in Surgically Treated Patients with Adenocarcinoma of the Ampulla of Vater
Source: Cancers (Basel). 2024 Aug 3;16(15):2756. doi: 10.3390/cancers16152756 (PMC11312068; doi:10.3390/cancers16152756)
Supplement: Supplementary file 1 [file cancers-16-02756-s001.zip › cancers-3123796-supplementary.pdf]

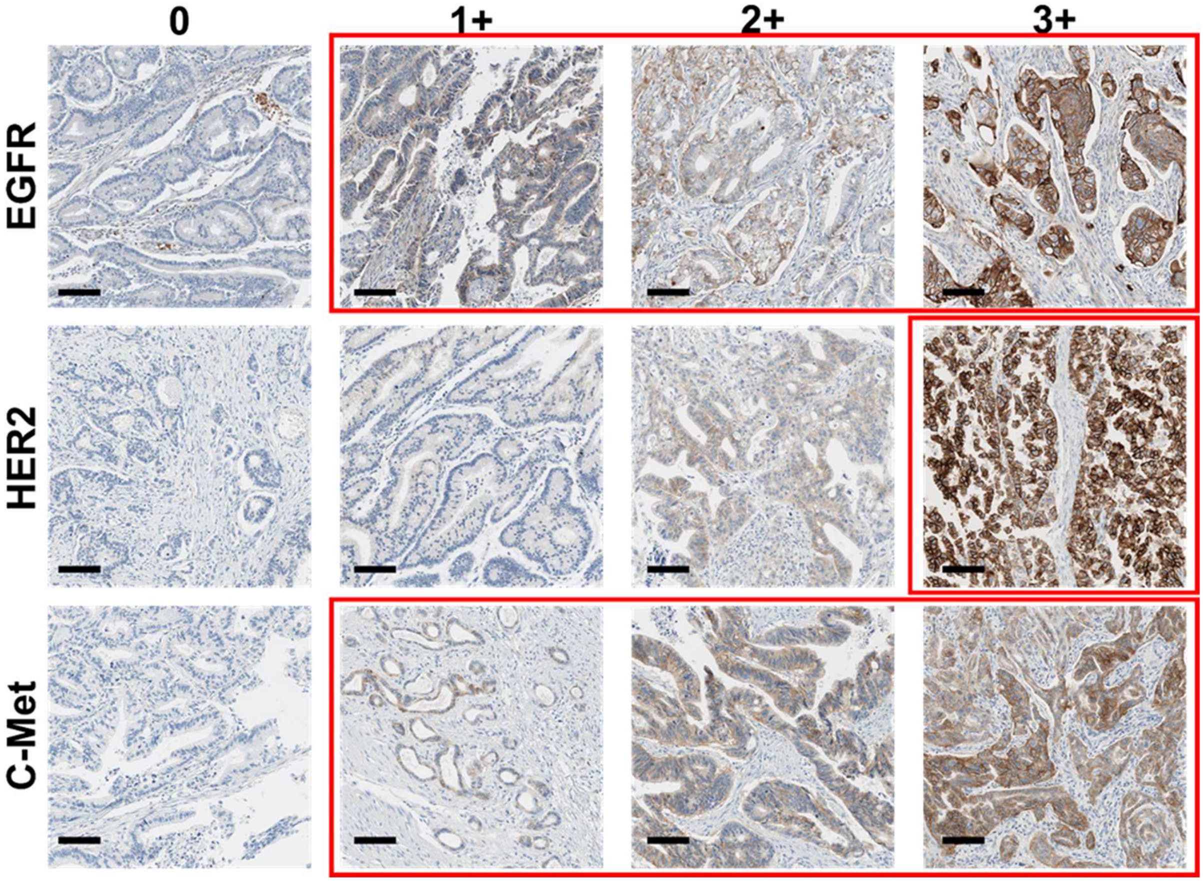

Figure S1. Representative images of EGFR, HER2, and c-Met with corresponding immunohistochemical scores ranging from 0 to 3+ (scale bar, 100 µm). The red box delineates the scores that were classified as positive for the purpose of this analysis.

Table S1. Comparison of clinicopathological factors among patients with ampulla of Vater carcinoma based on the expression of EGFR, HER2, and c-Met.

Variables	Total (n=87)	EGFR positive (n=76)	*p* value	HER2 positive (n=10)	*p* value	c-Met positive (n=43)	*p* value
**Age,** Median (Range)	65 (39-87)	65 (39-87)	0.567	67 (50-79)	0.378	65 (42-83)	0.600
**Gender, n (%)**							
Male	49 (56.3)	42 (55.3)	0.749	8 (80.0)	0.175	27 (62.8)	0.229
Female	38 (43.7)	34 (44.7)		2 (20.0)		16 (37.2)	
**Tumor size, cm, mean ± SD**	2.5 ± 1.1	2.4 ± 1.0	0.734	2.6 ± 1.1	0.625	2.3 ± 1.0	0.154
**Histologic grading, n (%)**							
Grade 1	15 (17.2)	10 (13.2)	**0.008**	1 (10.0)	1.000	8 (18.6)	0.739
Grade 2/3	72 (82.8)	66 (86.8)		9 (90.0)		35 (81.4)	
**Tumor Category, n (%)**							
T1-2	47 (54.0)	39 (51.3)	0.183	4 (40.0)	0.502	27 (62.8)	0.105
T3-4	40 (46.0)	37 (48.7)		6 (60.0)		16 (37.2)	

**Node Category, n (%)**							
N0	37 (42.5)	33 (43.4)	0.658	2 (20.0)	0.179	25 (58.1)	**0.004**
N1-2	50 (57.5)	43 (56.6)		8 (80.0)		18 (41.9)	
**TNM Stage*, n (%)**							
Stage I-II	38 (43.7)	34 (44.7)	0.601	2 (20.0)	0.175	26 (60.5)	**0.002**
Stage III-IV	49 (56.3)	42 (55.3)		8 (80.0)		17 (39.5)	
**EGFR expression, n (%)**							
Positive	76 (87.4)			8 (80.0)	0.608	41 (95.3)	**0.049**
Negative	11 (12.6)			2 (20.0)		2 (4.7)	
**HER2 expression, n (%)**							
Positive	10 (11.5)	8 (10.5)	0.608			4 (9.3)	0.739
Negative	77 (88.5)	68 (89.5)				39 (90.7)	
**c-Met expression, n (%)**							
Positive	43 (49.4)	41 (53.9)	**0.049**	4 (40.0)	0.739		
Negative	44 (50.6)	35 (46.1)		6 (60.0)			
**Lymphatic invasion, n (%)**							
No	37 (42.5)	30 (39.5)	0.192	4 (40.0)	1.000	23 (53.5)	**0.041**
Yes	50 (57.5)	46 (60.5)		6 (60.0)		20 (46.5)	

**Vascular invasion, n (%)**							
No	68 (78.2)	58 (76.3)	0.443	8 (80.0)	1.000	34 (79.1)	0.839
Yes	19 (21.8)	18 (23.7)		2 (20.0)		9 (20.9)	
**Perineural invasion, n (%)**							
No	64 (73.6)	56 (73.7)	1.000	9 (90.0)	0.279	32 (74.4)	1.000
Yes	23 (26.4)	20 (26.3)		1 (10.0)		11 (25.6)	
**Preoperative CA19-9 level, n (%)**							
Within normal (<40U/mL)	39 (44.8)	31 (40.8)	0.116	3 (30.0)	0.593	20 (46.5)	0.633
Above normal (≥40U/mL)	40 (46.0)	37 (48.7)		6 (60.0)		18 (41.9)	
Missing data	8 (9.2)	8 (10.5)		1 (10.0)		5 (11.6)	

EGFR epidermal growth factor receptor, *HER2* human epidermal growth factor receptor 2, *CA 19-9* carbohydrate antigen 19-9.

*According to American Joint Committee on Cancer 8th edition.
